# Spinous Process-Splitting Laminectomy Versus Conventional Laminectomy: A Short-Term Outcome Study

**DOI:** 10.7759/cureus.102648

**Published:** 2026-01-30

**Authors:** Ahmed K Basha, Hesham Radwan, Abdelmaksod Mohammed Mousa, Ahmed Nagaty, Abdelrahman Bakry, Eslam Hussein, Mohamed Ashraf Mahmoud, Mohammed Eid

**Affiliations:** 1 Neurological Surgery, Ain Shams University, Cairo, EGY; 2 Neurological Surgery, October 6 University, Giza, EGY

**Keywords:** back pain, laminectomy, lumbar spinal stenosis, oswestry disability index, spinous process-splitting

## Abstract

Background: Symptomatic lumbar spine stenosis (LSS) is a common spine degenerative disease often associated with back pain and neurogenic claudication pain. Laminectomy is a widely performed procedure in treating LSS, but it can lead to approach-related morbidity due to iatrogenic paraspinal muscle injury. Spinous process-splitting laminectomy (SPSL) is a muscle-preserving laminectomy technique that has been reported to provide superior functional outcomes and less postoperative pain.

Methods: This retrospective comparative study evaluated the conventional laminectomy (CL) and SPSL techniques regarding operative time, blood loss, postoperative pain, disability indices, and time to return to daily activities.

Results: A total of 27 patients were included in the study. The mean age of the participants was 62.5 ± 7.8 years, with 13 patients in the CL group and 14 patients in the SPSL group. The average intraoperative blood loss was less in the SPSL group (123.21 ± 54.12 ml) compared to the CL group (188.46 ± 79.46 ml). The mean operative time was 67.86 ± 33.27 min in the SPSL group and 87.69 ± 32.44 min in the CL group. The mean immediate postoperative low back pain on the Visual Analog Scale (VAS) at day one was 2.8 ± 0.6 for the CL group and 2.0 ± 0.0 for the SPSL group (p-value = 0.001). At six months, the mean postoperative low back pain on VAS was 0.77 ± 0.44 for the CL group and 0.21 ± 0.43 for the SPSL group (p-value = 0.012). The average Oswestry Disability Index (ODI) score at six months postoperatively was 2.21 ± 0.43 for the SPSL group and 4.38 ± 4.73 for the CL group (p-value = 0.001). The average time to return to normal activities of daily living was 2.07 ± 0.27 days in the SPSL group, much less than 6.77 ± 4.6 days in the CL group (p-value < 0.01).

Conclusion: SPSL represents a minimally invasive alternative to CL, with significant reductions in operative time and intraoperative blood loss. It also results in less postoperative pain and better functional outcomes, enabling an earlier return to daily activities.

## Introduction

Symptomatic lumbar spine stenosis (LSS) is a common spine degenerative disease, particularly in the elderly population. The prevalence of acquired LSS increases with advancing age, ranging from 20% in individuals less than 40 years to 47.2% in those in their seventh decade of life [1]. Entrapment of the cauda equina roots occurs due to osteoarthritic changes at the disc spaces, hypertrophy of the facet joints and ligaments surrounding the lumbar spinal canal, and subsequently acquired reduction in the diameter of the spinal canal. Symptomatic LSS is often associated with back pain, neurogenic claudication, and sometimes neurologic deficits [2]. It has a strong negative impact on the quality of life and physical status of those affected [3].

After conservative measures fail, a laminectomy is widely performed to treat LSS. However, approach-related morbidity resulting from iatrogenic soft tissue injury has become a major concern, which includes the paraspinal muscle injury from cauterization and extensive detachment from the posterior aspect of the lumbar spine [4].

Watanabe et al. were the first to describe the splitting of the spinous process as a tissue-preserving approach to performing a laminectomy in the management of LSS [5]. This procedure involves preserving midline osteo-ligamentous structures through bilateral retraction of the split spinous process and attached ligaments, which also helps preserve paraspinal muscle insertions. This typically results in less postoperative muscle atrophy and reduced paraspinal weakness. Additionally, this procedure also provides a wide visualization of the central canal and both lateral recesses [5].

In this report, we present a retrospective comparative study evaluating conventional laminectomy (CL) and spinous process-splitting laminectomy (SPSL) approaches for the decompression of lumbar spine canal stenosis, with an emphasis on their clinical and functional effects on postoperative pain and recovery.

## Materials and methods

Patient characteristics and setting

In this retrospective comparative study, we reviewed the charts of all patients who had symptomatic degenerative LSS without any radiological signs of instability and who underwent surgical decompression without fusion after failure of conservative management. All procedures were conducted at Ain Shams University-affiliated hospitals and October 6 University Hospital by only two surgeons - one at each institute - between January 2021 and January 2025. The learning curves of the two surgeons differed markedly, especially toward the later cases involving the SPSL procedure.

Patients who underwent surgical fusion, those with a history of previous spine surgery, and patients with peripheral vascular disease (vascular claudication pain), prolapsed disc disease, spinal tumors, fractures, or any radiological signs of spinal instability (except for grade 1 spondylolisthesis that appeared stable in dynamic plain radiographs) were excluded from our analysis.

Demographic data were extracted from patients’ charts. Preoperative clinical data, including preoperative walking distance, the presence or absence of radiculopathy, motor weakness, sensory hypoesthesia, the number of spinal levels affected, and the site of affection (central, lateral recess, or both) on preoperative magnetic resonance imaging (MRI), were also reported. The central canal stenosis was caused by hypertrophied ligamentum flavum, which reduced the anteroposterior diameter of the spinal canal, while the lateral recess stenosis was mainly caused by hypertrophied superior articular facets.

Operative technique

In both surgical techniques (CL and SPSL), a midline skin incision was made after lumbar spine level identification via fluoroscopy until reaching the lumbar fascia.

In the CL procedure, the paraspinal muscles were detached subperiosteally from the spinous process using monopolar cautery. The spinous process and the caudal part of the lamina were then resected using bone rongeurs and Kerrison punch forceps until the attachment of the ligamentum flavum and the cranial defect in the ligament were visible. The ligament was removed, and further bony decompression of the spinal canal was dependent on preoperative imaging. The lateral recess was also decompressed in necessary cases by undermining the hypertrophied superior articular facets.

In the SPS technique, before surgery, the vertical length of the spinous process was measured on preoperative imaging. Intraoperatively, the tip of the spinous process was freed and split into two halves using a chisel, marking it with a sterile marking pen to represent the preoperative length. The spinous process was then detached from the base of the lamina using a Cobb spinal elevator, with the paraspinal muscles still attached to each half, which were retracted to one side using hinged mastoid self-retaining retractors. The ligamentum flavum was then identified, opened, and resected. The remaining surgical steps were the same as in the CL procedure. At the end of the procedure and after adequate decompression of the neural structures, the retracted two halves of the split spinous process were readapted and fixed using polydioxanone (PDS) monofilament absorbable suture.

Closure in the CL procedure encompassed watertight fascial closure, and in both surgical techniques, subcutaneous tissue and skin closure were performed using surgical staples. A sub-fascial drain was used in cases of observed intraoperative bleeding tendencies.

Intraoperative findings

Average operative time, average intraoperative blood loss, and operative complications for both surgical approaches were reported.

Postoperative pain and functional outcomes

For assessment of postoperative clinical outcomes, we used the Visual Analog Scale (VAS) for back pain and leg pain immediately after complete patient recovery on postoperative day one and at six months postoperatively [6]. For assessment of postoperative functional outcomes, we used the Oswestry Disability Index (ODI), specifically the Oswestry Low Back Disability Questionnaire (the validated Arabic version) [7].

Functional assessments were conducted one and six months after surgery. Time to return to work (light duty work) and walking distance without resting before and after surgery at six months were also reported. Patients with a follow-up period of less than six months were excluded from our analysis.

Statistical analysis

All statistical analyses were performed using Microsoft Excel version 18.2110.13110.0 (Microsoft Corp., Redmond, USA) and IBM SPSS Statistics version 26 (IBM Corp., Armonk, USA). For the numerical variables, the independent t-test was used for normally distributed data, while the Mann-Whitney U test was used for non-normally distributed data. For categorical variables, the chi-square test was used to compare the subgroups. A p-value of less than 0.05 was considered statistically significant.

## Results

Our retrospective chart review revealed a total of 27 patients. Of these, 16 were male and 11 were female patients who met our inclusion criteria. The mean age of the study group was 62.5 ± 7.8 years (Table 1).

**Table 1:** Demographics and patient characteristics C: central canal; L: lateral recess

Parameter	Study groups (N = 27)
		Male, n (%)	16 (59.3%)
		Female, n (%)	11 (40.7%)
Male:female ratio	1.6:1.1
Age (years), mean ± SD	62.5 ± 7.8
Site of stenosis	
C, n (%)	5 (18.5%)
L, n (%)	7 (25.9%)
Both C&L, n (%)	15 (55.6%)
Number of levels operated	
1, n (%)	9 (33.3%)
2, n (%)	14 (51.9%)
3, n (%)	4 (14.8%)
Preoperative walking distance (meters), mean ±SD	100 ± 51.9
Preoperative radiculopathy	
Yes, n (%)	20 (74.1%)
No, n (%)	7 (25.9%)
Preoperative motor weakness	
Yes, n (%)	5 (18.5%)
No, n (%)	22 (81.5%)
Preoperative sensory hypoesthesia	
Yes, n (%)	8 (29.6%)
No, n (%)	19 (70.4%)

There were 13 patients in the CL group and 14 patients in the SPSL group. The average preoperative walking distance was 92.3 ± 57.2 meters in the CL group and 107.1 ± 47.5 meters in the SPSL group, and the difference between the two groups was statistically nonsignificant (p-value > 0.05) except for preoperative radiculopathy. The number of affected levels in each group is shown in Table 2.

**Table 2:** Differences in characteristics between the CL and SPSL groups ^a^ chi-square test; ^b^ independent t-test; * p-value significant if < 0.05 CL: conventional laminectomy; SPSL: spinous process-splitting laminectomy; C: central canal; L: lateral recess

Parameter	CL group (N = 13)	SPSL group (N = 14)	Test of significance	P-value
Male, n (%)	8 (61.5%)	8 (57.1%)	ꭕ^2^ = 0.054 ^a^	0.816
Female, n (%)	5 (38.5%)	6 (42.9%)		
Male:female ratio	1.6:1	4:3		
Age (years), mean ± SD	64.4 ± 8.5	60.8 ± 7.1	t = 1.21 ^b^	0.24
Site of stenosis				
C, n (%)	2 (15.4%)	3 (21.4%)	ꭕ^2^ = 2.097 ^a^	0.351
L, n (%)	2 (15.4%)	5 (35.7%)		
Both C&L, n (%)	9 (69.2%)	6 (42.9%)		
Number of levels operated				
1, n (%)	6 (46.2%)	3 (21.4%	ꭕ^2^ = 2.141 ^a^	0.343
2, n (%)	5 (38.5%)	9 (64.3%)		
3, n (%)	2 (15.4%)	2 (14.3%)		
Preoperative average walking distance (meters), mean ± SD	92.3 ± 57.2	107.1 ± 47.5	t = 0.736 ^b^	0.469
Preoperative radiculopathy				
Yes, n (%)	12 (92.3%)	8 (57.1%)	ꭕ^2^ = 4.731 ^a^	0.03 *
No, n (%)	1 (7.7%)	6 (42.9%)		
Preoperative motor weakness				
Yes, n (%)	1 (7.7%)	4 (28.6%)	ꭕ^2^ = 2.072 ^a^	0.15
No, n (%)	12 (92.3%)	10 (71.4%)		
Preoperative sensory hypoesthesia				
Yes, n (%)	4 (30.8%)	4 (28.6%)	ꭕ^2^ = 0.16 ^a^	0.901
No, n (%)	9 (69.2%)	10 (71.4%)		

**Figure 1:**
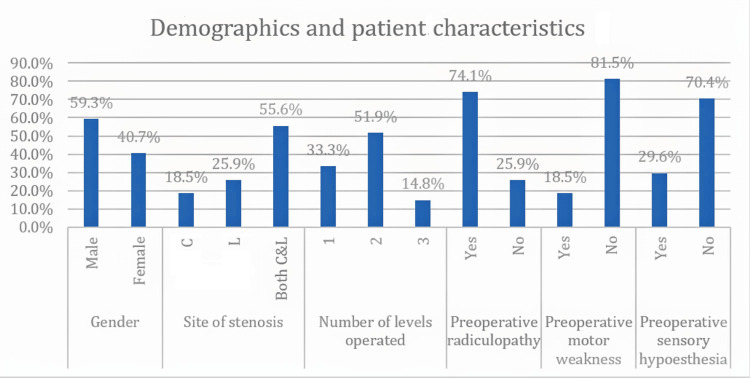
Demographics and patient characteristics of the whole cohort C: central canal; L: lateral recess

Twelve patients (92.3%) had preoperative radiculopathy in the CL group and eight patients (57.1%) in the SPSL group. Preoperative motor weakness was present in one patient (7.7%) in the CL group and in four patients (28.6%) in the SPSL group. Preoperative sensory hypoesthesia was found in four patients (30.8%) in the CL group and in four patients (28.6%) in the SPSL group (Table 2, Figure 1). The difference between the two groups regarding demographics, imaging characteristics, number of operated levels, and preoperative walking distance was statistically nonsignificant (p-value > 0.05) (Table 2, Figure 1). All our patients in both groups had neurogenic claudication as one of the presenting symptoms.

The average intraoperative blood loss was less in the SPSL group (123.21 ± 54.12 ml) compared to the CL group (188.46 ± 79.46 ml). Adjustment for the same number of affected levels was unreliable due to the small sample size. Additionally, the mean operative time was 67.86 ± 33.27 minutes in the SPSL group and 87.69 ± 32.44 minutes in the CL group (Table 3).

**Table 3:** Intraoperative blood loss and operative time ^a^ Mann-Whitney U test; * p-value significant if < 0.05 CL: conventional laminectomy; SPSL: spinous process-splitting laminectomy

Parameter	CL group (N = 13)	SPSL group (N = 14)	Test of significance	P-value
Operative time (min), mean ± SD	87.69 ± 32.44	67.86 ± 33.27	Z = 2.05 ^a^	0.043 *
Blood loss (ml), mean ± SD	188.46 ± 79.46	123.21 ± 54.12	Z = 2.29 ^a^	0.025 *

We had one case of intraoperative unintended durotomy and possible nerve root injury in the CL group. This patient developed postoperative lower extremity hypoesthesia that affected her early postoperative scores and delayed her return to normal activities of daily living. Although this hypoesthesia improved at the six-month follow-up, it did not resolve completely, impacting the late scores for the lower extremities.

We also had two patients with superficial wound infections, one in each group, which were managed conservatively with antibiotics and frequent wound dressing. No other complications were documented.

Mean immediate postoperative low back pain on the VAS at day one was 2.8 ± 0.6 for the CL group and 2.0 ± 0.0 for the SPSL group (p-value = 0.001). However, mean postoperative lower extremity pain for the same period on the VAS was 1.62 ± 1.45 for the CL group and 0.29 ± 0.73 for the SPSL group (p-value = 0.022). This difference may be explained by the patient who had intraoperative unintended durotomy and possible nerve root injury in the CL group.

The average postoperative walking distance in meters was 292.3 ± 101.7 for the CL group and 500 ± 103.8 for the SPSL group (p-value < 0.001), compared to 92.3 ± 57.2 and 107.1 ± 47.5 meters for the same groups preoperatively (p-value > 0.05) (Table 2, Table 4). One explanation for the difference in postoperative walking distance between the two approaches could be the patient who experienced intraoperative unintended durotomy and possible nerve root injury in the CL group, which likely affected postoperative scores.

**Table 4:** Immediate postoperative and follow-up clinical and functional characteristics ^a^ Mann-Whitney U test; ^b^ independent t-test; * p-value significant if < 0.05; ** p-value highly significant if < 0.001 LBP: low back pain; LE: lower extremity; ODI: Oswestry Disability Index; VAS: Visual Analog Scale; CL: conventional laminectomy; SPSL: spinous process-splitting laminectomy

Parameter	CL group (N = 13)	SPSL group (N = 14)	Test of significance	P-value
Mean LBP on VAS				
Immediate postoperatively, day 1 (mean ± SD)	2.8 ± 0.6	2 ± 0	t = 4.629 ^b^	0.001 *
At 6-month follow-up (mean ± SD)	0.77 ± 0.44	0.21 ± 0.43	Z = 2.83 ^a^	0.012 *
Mean LE pain on VAS				
Immediate postoperatively, day 1 (mean ± SD)	1.62 ± 1.45	0.29 ± 0.73	Z = 2.653 ^a^	0.022 *
At 6-month follow-up (mean ± SD)	0.85 ± 0.8	0.14 ± 0.36	Z = 2.608 ^a^	0.025 *
Average ODI				
At 1-month follow-up (mean ± SD)	8.77 ± 8.01	5.21 ± 0.8	Z = 3.362 ^a^	0.002 *
At 6-month follow-up (mean ± SD)	4.38 ± 4.73	2.21 ± 0.43	Z = 3.368 ^a^	0.001 *
Postoperative walking distance in meters (mean ± SD)	292.31 ± 101.7	500 ± 103.8	t = 5.245 ^b^	< 0.001 **
Average time to return to normal activities of daily living in days (mean ± SD)	6.77 ± 4.6	2.07 ± 0.27	Z = 4.676 ^a^	< 0.01 *

The average time to return to normal activities of daily living was 2.07 ± 0.27 days in the SPSL group, much less than the CL group (6.77 ± 4.6 days) (p-value < 0.01) (Table 4).

## Discussion

Degenerative lumbar stenosis is a common neurosurgical condition that, when symptomatic, is usually treated with simple decompressive laminectomy in the absence of any signs of instability. Fusion is sometimes needed in 5-10% of cases that are associated with degenerative scoliosis, degenerative spondylolisthesis, or pars fractures [8]. However, back pain sometimes remains unchanged or even increases post-laminectomy, qualifying as failed back surgery syndrome (FBSS) in around 30% of patients [9].

One of the major causes of post-laminectomy syndrome is paravertebral muscle dissection and retraction, which interrupts nerve and blood supply, causing muscle atrophy, postoperative muscle fibrosis, and back pain. These changes are common in the CL procedure due to marked paravertebral muscle disruption [10]. The SPSL approach aims to decrease paravertebral muscle dissection and degeneration. This is achieved by splitting the spinous process and separating both sides with the muscles attached to each side without cauterization of the paraspinal muscles and their nerve and blood supply [11].

Preservation of paraspinal muscles and posterior supporting structures has a significant impact on decreasing postoperative back pain and enhancing functional recovery, as indicated by several studies [5,12-14]. 

In our cohort, the mean immediate postoperative low back pain on the VAS was lower in the SPSL group, with a mean score of 2 ± 0, compared to 2.8 ± 0.6 in the CL group. This difference was statistically significant. Additionally, there was a statistically significant difference in low back pain VAS scores at the six-month follow-up between the SPSL and CL groups. This was reflected in both the average ODI at one and six months and the average time to return to normal activities of daily living, which were shorter in the SPSL group compared to the CL group (p-value < 0.01).

The SPSL approach is considered one of the minimally invasive methods, with less intraoperative blood loss and reduced operative time. These two factors are particularly crucial in the elderly population with a history of preoperative blood thinner use [15].

Operating in the prone position increases intra-abdominal and intrathoracic pressures and decreases cardiac compliance. These changes can elevate the risk of postoperative delirium due to reduced cerebral blood perfusion and increase the risk of deep venous thrombosis due to decreased venous flow from the lower extremities [16,17]. In our series, SPSL had less operative time and reduced intraoperative blood loss compared to the CL procedure, which is a desirable factor that may lower the possible risks associated with prolonged surgeries in the prone position.

The present study also shows that the mean postoperative low back pain on the VAS was lower for the SPSL group compared to the CL group in our series. This is consistent with findings in the literature regarding postoperative pain. Voglis et al. reported decreased postoperative pain and subsequently reduced analgesic use [13].

Faster postoperative recovery, with less postoperative pain and early mobilization, is a cornerstone of enhanced recovery after surgery (ERAS) and is generally encouraged after spine surgeries, especially in the elderly population, who often have multiple comorbidities that pose risks during the perioperative period. From the results of our study, we believe that SPSL enhances early postoperative recovery, aligning with recent trends toward ERAS [18].

Watanabe et al. were the first to introduce the SPSL approach as a minimally invasive alternative to CL [5]. In their original study, they demonstrated superior functional outcomes of SPSL using the Japanese Orthopedic Association (JOA) score compared to CL at two years. Our study showed consistent findings with previously reported results, with superior functional outcomes represented by a mean ODI score of 2.21 ± 0.43 compared to 4.38 ± 4.73 for SPSL and CL procedures, respectively, at the six-month follow-up [5].

However, these findings are contradicted by other studies that found no statistically significant differences between the two groups regarding functional outcomes and neurogenic claudication at three-month and one-year follow-ups, respectively [13,19].

In our series, there was a statistically significant difference in postoperative lower extremity pain in favor of the SPSL group (Table 4). We believe that both procedures are equal in their ability to relieve radiculopathy and neurogenic claudication and improve postoperative walking distance. The aforementioned difference in our cohort could largely be attributed to a higher number of preoperative radiculopathy patients in the CL group and the presence of a case of possible nerve root injury with persistent postoperative radiculopathy and hypoesthesia in the CL group, which affected the postoperative scores, especially given the small number of included cases in each group.

Limitations of the study

Selection bias is one of the inherent biases of retrospective studies; however, in our study, this was partially mitigated by including all patients who met our inclusion criteria. Most of the patients included during the first two years of the study period underwent the CL procedure, with a subsequent shift toward SPSL in almost all patients, except for one, during the last two years of the study period.

Additionally, our study had a relatively small sample size, which is reflected in the reduced statistical power of some parameters and makes the study more vulnerable to the augmented effects of outliers, as observed in our findings. 

Furthermore, there was a larger number of preoperative radiculopathy patients in the CL group compared to the SPSL group, which may have affected the postoperative results and could have been avoided by performing a multivariate analysis.

Another limitation was the short-term follow-up of our study. However, this does not strongly reflect on our primary outcomes (pain and functional outcomes), as they represent short-term outcomes. Moreover, pain and functional assessments in case of long-term follow-up could be affected by several confounders, such as adjacent segment stenosis or even instability, which may falsely impact our results.

## Conclusions

SPSL represents a minimally invasive alternative to CL. This approach significantly reduces operative time and intraoperative blood loss, which enhances postoperative recovery. It also results in less postoperative pain and better short-term functional outcomes, allowing for an earlier return to daily activities. However, these conclusions are preliminary and should be validated by more well-structured, prospective randomized studies. Further prospective, long-term studies are needed to address long-term outcomes and possible side effects, particularly instability, to provide a proper comparison with CL procedures.
